# HIV treatment outcomes among formerly incarcerated transitions clinic patients in a high prevalence setting

**DOI:** 10.1186/s40352-018-0074-5

**Published:** 2018-09-17

**Authors:** Mariya I. Masyukova, David B. Hanna, Aaron D. Fox

**Affiliations:** 1 Department of Family and Social Medicine, Montefiore Medical Center/ Albert Einstein College of Medicine, Bronx, New York, USA; 2 Department of Epidemiology and Population Health, Albert Einstein College of Medicine, Bronx, New York, USA; 3 Department of Medicine, Division of General Internal Medicine, Montefiore Medical Center/ Albert Einstein College of Medicine, Bronx, New York, USA

**Keywords:** HIV, Incarceration, Re-entry, Primary care, Retention in care, Transitions clinic

## Abstract

**Background:**

Incarceration disproportionately affects people living with HIV/AIDS. When people are released from jail or prison, they face multiple barriers to HIV care, and those who do engage in care may have suboptimal HIV treatment outcomes. A limited number of studies have investigated HIV treatment outcomes among people who have been released from incarceration.

**Methods:**

We conducted a retrospective cohort study comparing HIV viral load (VL) suppression and retention in care 12 months after entry into care among patients of a post-incarceration Transitions Clinic (TC) and a comparison group who received HIV care in the same community. Of 138 participants, 38 TC patients were matched to 100 non-TC controls based on age, race/ethnicity, gender, and date of HIV care entry.

**Results:**

There was no significant difference in clinical study outcomes between TC and non-TC patients: 63% vs. 67% (*p* = 0.67) were retained in care and 54% vs. 63% (*p* = 0.33) had suppressed VL at 12 months. After adjusting for substance use disorder and viral load suppression at the start of treatment, the odds ratio of TC patients’ 12-month retention was 0.60 (95% CI 0.25–1.49) and VL suppression was 0.44 (95% CI 0.16–1.23) compared with non-TC patients.

**Conclusions:**

Our findings show HIV care outcomes for patients at a post-incarceration Transitions Clinic that are similar to those of community-based comparison patients. The transitions clinic model, which provides medical, behavioral health, and supportive services to formerly incarcerated people, may be an effective model of care for this population; however, more scholarship is needed to quantify the components most effective in supporting retention in care and viral load suppression.

## Background

One in seven Americans with human immunodeficiency virus (HIV) infection pass through a correctional facility each year. (Spaulding et al., 2009) Reflecting the disproportionate disease burden, HIV prevalence among the formerly incarcerated is several-fold higher than that of the general population (1.3% vs. 0.4%). (Maruschak et al., 2015) Life-sustaining treatment for HIV infection should be available in correctional facilities; however, clinical indicators, including HIV viral load and CD4 cell count, often worsen following release from incarceration. (Springer et al., 2004; Stephenson et al., 2005; Westergaard et al., 2011) During this reentry period, poor access to antiretroviral treatment (ART), often exacerbated by housing instability, unemployment, and resumption of substance use, may explain worsening HIV viral load and CD4 count. (Althoff et al., 2013; Baillargeon et al., 2009; Binswanger et al., 2011; Cooke, 2004; Dennis et al., 2015; Lanier & Paoline, 2005; Rich et al., 2001).

Linkage to medical care after incarceration has been an important area of focus in efforts to improve access to HIV care. (Flanigan, 2013; Liau et al., 2013; Westergaard et al., 2013; Zaller et al., 2008) However, data regarding long-term treatment outcomes beyond the immediate post-release period are fragmented, outcomes that have been quantified are suboptimal, and health disparities likely remain. (Frank et al, 2014; Iroh et al., 2015; Loeliger et al., 2018; Swan, 2016; Wohl & Rosen, 2018) Studies that have reported on post-release HIV-related outcomes have focused on viral load suppression using data from research visits in clinical trials, correctional facility records for people who were released and re-incarcerated, or public health surveillance, where there was uncertainty whether participants have been retained in clinical care or whether laboratory data came from acute episodes of care. (Baillargeon et al., 2010; Loeliger et al., 2018; Palepu et al., 2004; Wohl et al., 2017) “Real-life” clinical data, including measures of retention in care, could contribute to the growing body of literature on post-release HIV outcomes.

Transitions clinics, where patients receive medical appointments soon after release from incarceration and clinicians have experience providing medical care to patients with criminal justice involvement, are an emerging linkage model. (Fox et al., 2014; Wang et al., 2010) Providing case management and identifying and addressing medical and mental health needs are associated with improved HIV-related outcomes. (Loeliger et al., 2018) However, even after linkage to care, formerly incarcerated patients may continue to experience disparities in HIV-related outcomes. More detailed data on clinical outcomes could help improve interventions that target linkage, retention in care, and viral load suppression. (Haley et al., 2014; Wohl & Rosen, 2018).

In this retrospective cohort study, we investigated HIV treatment outcomes among formerly incarcerated individuals who received care in a transitions clinic and compared these outcomes to a matched sample of individuals who initiated HIV care in the same low-income urban community. Because of the unique risk factors associated with a history of incarceration, we hypothesized that transitions clinic patients would have poorer HIV treatment retention and virologic outcomes. However, the communities to which many formerly incarcerated persons return also reflect many of the same risk factors for suboptimal HIV outcomes, regardless of incarceration history. (Clear, 2008; Freudenberg, 2001; Poundstone et al., 2004; Schnittker et al., 2011) We focused on outcomes among patients who initiated HIV care in order to identify unique treatment needs for formerly incarcerated individuals, which may help tailor models of care.

## Methods

This study was approved by the institutional review board of the Albert Einstein College of Medicine.

### Clinical settings

Three ambulatory sites from which the sample was drawn in the Bronx, NY included the Bronx Transitions Clinic (TC), a community health center (CHC), and an infectious disease clinic (IDC). The urban neighborhoods where the sites are located are comprised of largely minority populations (54–57% Hispanic/Latino, 25–39% African American), with 33–41% of people living below the poverty line and a higher HIV and substance use burden than the rest of New York City. (Olson et al., 2006b,Olson et al., 2006a).

The TC, a partnership between a nonprofit organization and the CHC, is located within the CHC facility. Patients, many of whom are HIV-positive, are referred to the TC on discharge from state prison or local jail. (The Osborne Association, 2015) A formerly incarcerated community health worker provides patient navigation services. Primary care physicians who provide care at the TC also practice at the CHC. Other services offered at the CHC, including social work, mental health, substance abuse treatment and specialty care, are available to TC patients. (Fox et al., 2014) The CHC is a federally qualified health center with general primary care and specialty practices on site.

The IDC, located in a neighboring area, is one of the largest HIV specialty clinics in the state. Its patients have access to multidisciplinary services, including nutritional counseling, case management, group programs, mental health services, and substance use disorder treatment. There are no services directly tailored to the needs of formerly incarcerated persons.

### Study population and data collection

Participants were adults (≥ 18 years old) with documented HIV infection who had at least one visit at the TC, CHC, or IDC between 8/1/09 and 12/31/13 and had at least one set of viral load and CD4 cell count values documented in the Electronic Health Record (EHR). We identified formerly incarcerated HIV-infected patients at the TC through an internal database. A comparison group of patients from the CHC and the IDC were identified through the HIV Clinical Cohort Database of the Einstein-Rockefeller-CUNY Center of AIDS Research, which contains data on all HIV-positive patients in the greater health network. (Hanna et al., 2016) The comparison group was matched to TC patients based on demographics (age ± 5 years, race/ethnicity, gender) and date of HIV care initiation (±6 months), a strategy similar to that in previously published research. (Wang, Wang, & Krumholz, 2013) Care initiation was included in the matching criteria to account for secular trends, and was defined as the first ever documented visit, the first visit for HIV–specific medical care following a new diagnosis, or a visit after 12 months without a visit (if multiple dates fit the last criterion, we chose the latest re-initiation visit after 12 months out of care). Clinical data were manually extracted from the EHR common to all sites and stored using a secure database management web application.

**Fig. 1:**
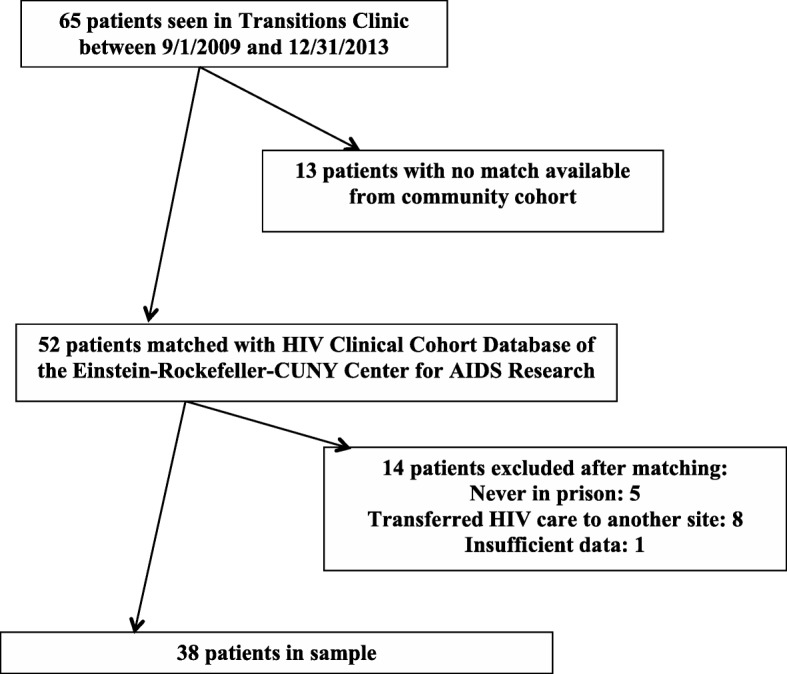
Disposition of Transitions Clinic Subjects

Of 65 HIV-positive TC patients initiating care in this time frame, 38 were matched to a comparison group of 1–3 CHC or IDC controls, depending on the availability of controls in the database that fulfilled matching criteria. The resulting sample comprised 38 TC patients and 100 non-TC patients (16 from CHC and 84 from IDC) (see Fig. 1). A documented history of incarceration in state or federal prison was necessary for inclusion in the TC sample and was verified using a public web-based database. (Inmate Population Information Search, 2015) Patients were excluded from the TC sample if they did not have a history of incarceration in state or federal prison or if they transferred care to a non-TC HIV provider after their initial TC visit. There were no known deaths during the study period. Outcomes data for the  four patients who were incarcerated within their 12-month follow-up were included in all analyses.

### Key measures

#### Exposure of interest

The main independent variable was site of care: patients at TC were compared with the demographically matched cohort at CHC and IDC.

#### Dependent variables

Primary outcomes were HIV VL suppression and retention in care 12 months after entry into care. Retention in care at 6 months was a secondary outcome. HIV VL suppression was a dichotomous variable defined as a value of < 75 copies/mL drawn between 180 and 360 days of the initial visit; if no results were documented in this period, VL was recorded as “not suppressed”. Retention in care at 6 months was defined as having at least two visits, separated by at least 90 days, within the 180-day period after care initiation, based on a previously published measure. (Althoff et al., 2013) Retention in care at 12 months required 6-month retention and one more visit between 180 and 360 days from treatment initiation (i.e. at least three visits total).

#### Covariates

HIV infection-related variables extracted from medical records included: recent (within 12 months of care initiation) HIV diagnosis, reported number of years after HIV diagnosis, being on antiretroviral treatment, risk factor for acquiring HIV (heterosexual sex, men having sex with men, injection drug use, blood transfusion), and whether HIV viral load was suppressed at care initiation. Comorbidities extracted from the medical records included the following: chronic illness, hepatitis C infection, substance use disorder (subdivided into opioid use disorder, alcohol use disorder, cocaine use disorder), diagnosis of psychiatric illness, chronic non-malignant pain, current tobacco smoking, receiving buprenorphine maintenance treatment, and receiving chronic opioid analgesics (for more than 3 consecutive months). We defined “chronic illness” as any diagnosis of chronic non-infectious disease (e.g., diabetes and hypertension). Substance use variables referred to past or current disordered use of heroin, cocaine, amphetamines, illicit benzodiazepines, or alcohol use disorder (see Table 1). “Psychiatric illness” included documented Axis I diagnoses as characterized by the DSM-IV. “Chronic non-malignant pain” was defined as clinically documented pain lasting longer than 12 weeks due to conditions other than malignancy (e.g. osteoarthritis, neuropathy).

**Table 1:** Patient demographic and clinical characteristics, by site of care

Covariate		Comparison group (*n* = 100)	Transitions (*n* = 38)	*p*-value^1^
*Demographics*				
Age: Mean (S.D.)		49 (9)	49 (9)	0.98
Male gender		90 (90%)	35 (92%)	0.71
Race	Black/African-American	52 (52%)	17 (45%)	0.09
	Hispanic/Latino	46 (46%)	17 (45%)	
	Other^2^	2 (2%)	4 (11%)	
*HIV variables*				
VL suppressed at care initiation		29 (29%)	22 (58%)	< 0.01
Recent HIV diagnosis^3^		19 (18%)	2 (5%)	0.04
Reported years diagnosed with HIV^4^ (S.D.)		11.0 (9.5)	14.8 (8.7)	0.03
On ART during follow-up		90 (90%)	37 (97%)	0.18
HIV risk factor	Heterosexual	58 (58%)	20 (53%)	0.57
	MSM^5^	33 (32%)	2 (5%)	< 0.01
	Injection drug use	12 (12%)	20 (53%)	< 0.01
	Transfusion	1 (1%)	0 (0%)	N/A
	Unknown	1 (1%)	4 (11%)	< 0.01
*Comorbidities*				
Chronic illness^6^		67 (67%)	24 (63%)	0.67
Hepatitis C infection		19 (19%)	25 (66%)	< 0.01
Current substance use disorder		24 (24%)	22 (58%)	< 0.01
Current opioid use disorder^7^		12 (12%)	21 (55%)	< 0.01
Current cocaine use disorder		5 (5%)	8 (21%)	< 0.01
Current alcohol use disorder		9 (9%)	5 (13%)	0.47
Psychiatric illness^8^		39 (39%)	20 (53%)	0.15
Chronic non-malignant pain^9^		39 (39%)	21 (55%)	0.09
Current smoking		41 (41%)	30 (79%)	< 0.01
Receiving buprenorphine maintenance treatment		2 (2%)	11 (29%)	< 0.01
Receiving prescription opioid analgesics^10^		21 (21%)	3 (8%)	0.08

^1^For statistical tests of bivariate association: t-test for continuous variables, chi-square for categorical variables, Fisher’s Exact test for categorical variables with expected cell size < 5

^2^“Other” includes white and mixed-race

^3^Within 12 months of care initiation

^4^HIV diagnosis date information was available for 131 patients total

^5^Sexual transmission: men who have sex with men

^6^Chronic non-infectious disease diagnosis: e.g. hypertension, hyperlipidemia, diabetes mellitus, etc.

^7^DSM-IV diagnosis of opioid dependence

^8^DSM-IV Axis I diagnoses, including: Major Depressive Disorder, Generalized Anxiety Disorder, Bipolar Disorder, Mood Disorder (Not Otherwise Specified), Post-Traumatic Stress Disorder, Panic Disorder, Schizophrenia, Schizoaffective Disorder, AIDS Dementia Complex

^9^Pain lasting > 12 weeks due to factors other than malignancy, e.g. intervertebral disc disease, osteoarthritis, or neuropathy

^10^Chronic pain as described above; HIV provider renews opioid analgesic prescription to be used daily for at least 3 continuous months

### Data analysis

We used tests of bivariate association to determine whether patients receiving care at the Transitions Clinic differed from patients at other sites in demographic and clinical variables and outcomes. To determine whether TC patients had worse HIV treatment outcomes than the comparison group, we used multivariable logistic regression. We developed one set of models for each outcome, with the outcome as the dependent variable and the site of care (TC vs. non-TC) as the main independent variable. Covariates ultimately included in the multivariable model differed by site of care (with *p* < 0.20 in bivariate analyses) and were deemed to be the most clinically relevant. The small sample size limited the number of covariates included in the final model.

We developed two regression models for each outcome variable:

Model 1: Unadjusted, with site of care (TC vs. non-TC) as the only predictor;

Model 2: Adjusted for substance use disorder and HIV VL suppression at the beginning of the study period.

Models with VL suppression as the outcome were limited to those patients who were on ART within the follow-up period (*n* = 127). All other models were run on the entire sample (*n* = 138). Stata software was used for all analyses (StataCorp. 2013. *Stata Statistical Software: Release 13*. College Station, TX: StataCorp LP.)

## Results

### Sample characteristics

The complete sample included 138 HIV-positive patients: 38 from the Transitions Clinic, 16 non-TC patients from the Community Health Center, and 84 patients from the Infectious Disease Clinic. Most (91%) were male with mean age 49 years (ranging from 25 to 68 years). Most were identified as African American (50%) or Hispanic (47%). On average, patients had been living with HIV for 12 (+/− 9) years, though 15% were diagnosed within 12 months of initiating care. The most commonly reported risk factors for HIV transmission were presumed heterosexual contact (57%), MSM contact (25%), and injection drug use (23%), which were not mutually exclusive. Sixty-seven percent of the sample had at least one chronic illness, 43% had documented psychiatric illness, and one third reported current or past substance use; 24% had been diagnosed with opioid use disorder, and nearly half reported suffering from chronic non-malignant pain.

### Transitions and comparison groups were clinically distinct

There were several differences between TC and non-TC patients (see Table 1). Fewer TC patients were recently diagnosed with HIV (5% vs. 18%, *p* < 0.05), and TC patients had lived with HIV longer (mean 15 vs. 11 years, *p* < 0.05). TC patients were more likely to report injection drug use as the initial HIV transmission risk factor (53% vs. 12%, *p* < 0.01), to have suppressed VL at care initiation (58% vs. 24%, *p* < 0.01), and to have current substance use disorder (58% vs. 24%, *p* < 0.01). There were no significant differences between groups in the prevalence of chronic disease or psychiatric illness.

### Retention in care was not significantly different between groups

There were no statistically significant differences in 6- and 12-month retention between TC and non-TC patients, with 76% of TC patients (vs. 79% non-TC) retained at 6 months and 63% (vs. 67% non-TC) retained at 12 months. In both unadjusted and adjusted logistic regression models, the odds of being retained in care were not significantly different between TC and non-TC patients. In the adjusted model, the odds of 6- and 12-month retention in care were nonsignificantly lower for TC patients, with OR = 0.72 (95% CI 0.26, 1.00) for 6-month retention and OR = 0.60 (95% CI 0.25, 1.49) for 12-month retention in care (see Table 2).

**Table 2:** Adjusted association of site of care with retention in care and virologic suppression

Logistic regression models, *N* = 138		
Outcomes	Model 1^a^: Unadjusted	Model 2^b^: adjusted
Odds of 12-month retention	0.84 (0.39, 1.84)	0.60 (0.25, 1.49)
Odds of 6-month retention	0.86 (0.35, 2.08)	0.72 (0.26, 1.00)
Odds of VL suppression^c^:	0.68 (0.31, 1.48)	0.44 (0.16, 1.23)

^*a*^ *Model 1: Unadjusted (site of care as the only covariate)*

^*b*^ *Model 2: Adjusted for suppressed viral load at care initiation and current substance use disorder*

^*c*^ *N = 127 (restricted to participants prescribed antiretroviral therapy)*

In the adjusted models, having suppressed HIV viral load at care initiation was significantly associated with both 12-month retention in care and viral load suppression. Current substance use was not significantly associated with either outcome. (see Table 3) There was no statistically significant difference in viral load suppression between TC and non-TC patients, with 54% of TC patients (vs. 63% non-TC, *p* = 0.33) having suppressed VL at 12 months. In both unadjusted and adjusted logistic regression models, the odds of viral load suppression at 12 months were non-significantly lower in TC vs. non-TC patients, with OR = 0.68 (95% CI 0.31, 1.48) in the unadjusted and 0.44 (95% CI 0.16, 1.23) in the adjusted model. (see Table 2).

**Table 3:** Factors associated with retention in care and virologic suppression at 12 months in adjusted model

Covariate	Odds ratio of 12-month retention (95% CI)	Odds ratio of having suppressed HIV viral load within 12 months (95% CI)
Site of care: Transitions Clinic	0.60 (0.25, 1.49)	0.44 (0.16, 1.23)
Suppressed VL at treatment initiation	2.76 (1.21, 6.28)	7.72 (2.90, 20.54)
Current substance use disorder	1.04 (0.46, 2.34)	0.46 (0.20, 1.13)

## Discussion

Our study describes unique treatment needs of formerly incarcerated HIV-positive patients. Despite particular vulnerabilities, once formerly incarcerated patients were linked to care, successful outcomes (similar to those of a demographically matched community cohort) were possible. HIV infection morbidity is inequitably distributed in communities to which formerly incarcerated people return, and we sought to describe HIV outcomes for formerly incarcerated patients at a Transitions Clinic in the context of their already vulnerable communities.

Other studies have demonstrated worsening HIV control after release from incarceration. One older study demonstrated that HIV treatment outcomes worsened following release from jail. (Springer et al., 2004) A more recent multi-site study of linkage to HIV care following release from jail demonstrated rates of 6-month retention in HIV care (50–63%) and viral load suppression (25%) that were lower than in our study, but the difference in source populations (i.e., jail vs. prison) makes direct comparison difficult. (Meyer et al., 2014; Spaulding et al., 2013) Among individuals who received a comprehensive discharge plan and case management following release from a New York City jail, fewer than one-third had a suppressed HIV viral load (< 400 copies/ml) at 6 months. (Teixeira et al., 2015) Regarding prison release, one large retrospective cohort study reported post-release linkage to care and viral load suppression using public health surveillance data, which documented that 40–73% of participants had a suppressed viral load at the time of linkage to community care depending on how soon after release they initiated care; however, this study did not report ongoing retention in care or viral load suppression post-linkage. (Loeliger et al., 2018) Other studies have evaluated linkage to care following prison release, but have not assessed long-term treatment outcomes. (Baillargeon et al., 2009; Devereux et al., 2002; Khawcharoenporn et al., 2013; Rich et al., 2001; Wohl et al., 2011; Zaller et al., 2008) Our study used manually extracted medical visit data to quantify treatment retention and reported ongoing viral load suppression after care initiation. Thus, our data fills a gap in the literature and may aid in the tailoring of HIV treatment delivery models to meet the needs of formerly incarcerated individuals.

Our measured outcomes were similar between formerly incarcerated TC patients and the community cohort. The odds of retention in care and viral load suppression were, as we expected, lower in the TC cohort, though this was not statistically significant. Though our study was not powered to detect non-inferiority, it is reassuring that larger differences did not exist in our sample. In a cross-sectional study of Veterans’ Administration patients, previously incarcerated persons were more likely to have a viral load > 500 copies/ml and CD4 count less than 200 cells/μL; however, this study’s exposure of incarceration included any prior incarceration, not solely recent release from prison. (Wang et al., 2015) In a cohort from British Columbia, any incarceration within 12 months of ART initiation was associated with lower ART adherence and viral load suppression; however, the cohort included participants who initiated ART prior to and during incarceration, whereas outcomes for participants who entered care post-release were not specified. (Palepu et al., 2004; Milloy et al., 2011) In a cohort of people who inject drugs who achieved virologic suppression on ART, incarceration during follow-up was associated with virologic failure. (Westergaard et al., 2011) Incarceration can cause disruptions in HIV care, but with appropriate linkage to care post-release, it is less clear whether an incarceration history continues to affect clinical care. Larger studies are necessary to confirm a more precise estimate of differences in HIV treatment outcomes between those with and without recent incarceration, as our results could reflect a true difference in outcomes suggesting a higher-risk, higher-need population.

Of note, more TC patients had chronic non-malignant pain and/or an opioid use disorder, which may have had a negative effect on outcomes. Substance use disorders are associated with poor HIV outcomes (Altice et al., 2010; Lucas, 2011) including among formerly incarcerated individuals. (Chitsaz et al., 2013; Wang et al., 2015) In our study, the use of buprenorphine maintenance treatment by about half of TC patients diagnosed with an opioid use disorder may have mitigated some of the effects of substance use, particularly opioid use. Other differences, such as parole requirements or stigma from criminal justice involvement, could have influenced HIV treatment outcomes, but we could not assess for these factors with our data.

Importantly, more TC patients than non-TC patients had a suppressed VL at initiation of care, which was highly associated with both VL suppression and retention in care at 12 months. This confirms our clinical observation that despite comorbidity and psychosocial risk, many formerly incarcerated individuals are highly engaged in HIV care during incarceration; when assured continued access to care in the community, they can maintain viral load suppression despite the challenges of community reentry. However, as we hypothesized and in line with prior research, there were a number of transitions clinic patients who entered care with a suppressed viral load but were unable to maintain viral load suppression. (Palepu et al., 2004).

Formerly incarcerated individuals’ unique strengths may be an underutilized resource in HIV care for this population. More research is needed that focuses on formerly incarcerated individuals’ positive resilience and protective factors, rather than relying on a deficiency-based frame. Prior qualitative studies suggest that supportive social relationships, facility with coping skills, support for resource navigation, and positive individual attitudes are factors contributing to resilience, and, ultimately, improved clinical outcomes. (Bracken et al , 2015; Dennis et al., 2015; Fuller et al., 2018) Recent quantitative findings suggest a correlation between medical complexity and short-term healthcare utilization; persons with comorbid conditions may have skills or supports that could also be helpful to to less “medically complex” patients, or that the medical community at large must shift conventional risk assessment paradigms to include incarceration as a relevant health risk. (Loeliger et al., 2018).

We also found some similarities between the two study groups. Though it has been previously documented that formerly incarcerated persons have a greater burden of mental illness and chronic non-infectious diseases, (Healthcare in New York Prisons 2004–2007, 2009; Springer et al., 2011) these differences were not reflected in our study sample consisting of HIV-positive patients who initiated clinical care. In our sample, 15% of the non-TC patients also had a history of prior incarceration, unsurprising given that participants were almost exclusively people of color living in a large U.S. city during the era of mass incarceration. These similarities reflect the high burden of mental, physical, and social illness affecting the source population, (Alexander, 2012) and, in turn, suggest that systemic and social change should be a public health priority.

The TC model may be effective in meeting the multifaceted needs of formerly incarcerated patients, but the efficacy of specific model components will require further inquiry. Most linkage interventions have focused on case management and facilitating entrance into care, (Nunn et al., 2010) but little is known regarding outcomes of case management beyond a short time frame. (Wohl et al., 2017) Competing demands that may determine survival (e.g. housing, employment, social support) also affect adherence to care for the formerly incarcerated. (Dennis et al., 2015) Our study did not quantify the impact of competing needs following linkage, but this is an important direction for future research. The transition of care from a controlled environment like prison to a community setting with different requirements for HIV treatment adherence may also require specific education or supports directed at self-management. More work is needed to understand best practices for helping formerly incarcerated individuals navigate the health care system, adhere to medications, and maintain retention in HIV care.

The strengths of our study include the unique sample of patients, matching based on initiation of care to account for secular trends, and inclusion of granular clinical information that may not be available in epidemiologic studies. Still, this work is preliminary and has multiple limitations. Our sample size limited power to detect small differences in HIV outcomes that could have been clinically meaningful and limited the number of covariates we could analyze in our final model. Our matching accounted for demographic characteristics, but there are other potential unmeasured confounders, such as stigma, housing, employment status, and social support, that likely had a meaningful effect on outcomes. Importantly, 15% of persons in the comparison group had a history of incarceration, as verified by the public database; though this reflects real-life complexity of the patient population, it also presents a potential confounder, which limits conclusions attributed to incarceration history. Furthermore, other than a peer community health worker, services available at the transitions clinic and non-transitions sites were similar. Larger studies are necessary to confirm our findings, but this area of inquiry is useful to inform practice and policy regarding the complex challenges that formerly incarcerated persons face.

## Conclusion

Formerly incarcerated HIV-positive patients receiving care in a transitions clinic were more likely to have substance use, chronic pain, and to have acquired HIV through injection drug use compared to demographically matched patients initiating care in other HIV care settings. However, patients at the transitions clinic had similar outcomes related to HIV viral load suppression and retention in care after 12 months. Developing robust clinical programs, like transitions clinics, which include mental health services, substance use disorder treatment, and peer support, may mitigate some risk for formerly incarcerated individuals. Given the similarities between transitions clinic patients and their HIV-positive peers in the community, broader social change addressing racial and socioeconomic health disparities should be a public health priority.

## Abbreviations

AIDS: Acquired Immunodeficiency Syndrome
ART: Antiretroviral therapy
CHC: Community health center
DSM: Diagnostic and Statistical Manual
EHR: Electronic health record
HIV: Human immunodeficiency virus
IDC: Infectious disease clinic
MSM: Men who have sex with men
TC: Transitions clinic
VL: Viral load
